# Microstructures and High-Temperature Mechanical Properties of Inconel 718 Superalloy Fabricated via Laser Powder Bed Fusion

**DOI:** 10.3390/ma17153735

**Published:** 2024-07-28

**Authors:** Nan Li, Changshun Wang, Chenglin Li

**Affiliations:** 1 AECC Beijing Institute of Aeronautical Materials, Beijing 100095, China; 2 School of Power and Mechanical Engineering, Wuhan University, Wuhan 430072, China; 2022102080012@whu.edu.cn

**Keywords:** laser powder bed fusion, superalloy, high-temperature tensile, high-temperature nanoindentation

## Abstract

The Inconel 718 superalloy demonstrates the potential to fabricate high-temperature components using additive manufacturing. However, additively manufactured Inconel 718 typically exhibits low strength, necessitating post-heat treatments for precipitate strengthening. This study investigated the microstructures and mechanical properties of the Inconel 718 superalloy fabricated via laser powder bed fusion. The room-temperature and high-temperature tensile properties of the Inconel 718 alloy samples following various post-heat treatments were evaluated. The results indicate that the as-built samples exhibited columnar grains with fine cell structures. Solution treatment resulted in δ phase formation and grain recrystallization. Subsequent double aging led to finely distributed nanoscale γ′ and γ″ particles. These nanoscale particles provided high strength at both room and high temperatures, resulting in a balanced strength and ductility comparable to the wrought counterpart. High-temperature nanoindentation analyses revealed that the double-aging samples exhibited very high hardness and low creep rates at 650 °C.

## 1. Introduction

Nickel-based superalloys are extensively utilized for fabricating components operating at high temperatures, such as heat-resistant parts and turbine blades, due to their exceptional strength, stability, and fatigue resistance at temperatures up to 1000 °C [1,2]. Inconel 718 (or IN718) stands out as a typical superalloy renowned for its high-temperature strength and overall performance [3]. Unlike solid-solution hardened superalloys, like IN625 or Hastelloy X, IN718 is an age-hardened Ni-based superalloy. Its microstructure primarily comprises the γ-phase, γ′ (Ni = (Al, Ti, Nb), L_1_2), (Ni_3_Nb, DO_22_), δ-phase (Ni_3_Nb, DOa), and carbides [4]. Among these constituents, γ′ and γ″ precipitates, typically in the size range of tens of nanometers, serve as the principal strengthening phases.

Additive manufacturing (AM) techniques, such as laser powder bed fusion (L-PBF), and laser-directed energy deposition (L-DED), are increasingly employed for fabricating complex metallic materials, including titanium alloys and superalloys [5,6,7,8,9,10,11,12]. AM offers distinct advantages, such as ease and high accuracy in manufacturing components with intricate shapes and geometries [13]. The ultimate quality and mechanical performance of the fabricated parts strongly rely on the AM process and subsequent treatments. The key AM parameters such as laser power, scanning speed, layer thickness, and energy density significantly influence these mechanical properties [14].

L-PBF generally results in the formation of a fine-grained structure and fine cells in IN718 compared to its wrought counterpart; however, due to the limited presence of precipitates (γ″), its strength is initially lower than that of the aged state. For example, the L-PBF as-built IN718 typically exhibits a strength level of less than 1 GPa, while the strength of the samples after aging can exceed 1400 MPa, thanks to the formation of finely dispersed precipitates during the aging process. Consequently, various post-heat treatments are extensively utilized to improve the mechanical properties of LPBF-built IN718. Stress-relieving (SR), hot isostatic pressing (HIP), solution treatment (ST) or homogenization annealing, and aging (single or double-step) are the most commonly used heat treatments. The laser-based AM processes typically generate significant residual stresses that can compromise the mechanical properties. These residual stresses can be alleviated through SR treatments conducted at sufficiently high temperatures and extended holding times. HIP is commonly employed to decrease the porosity of the additive manufactured (AM) parts. During the HIP processes, grain recrystallization and grain coarsening may occur. Previous studies have indicated that the porosity in IN718 can be reduced from 0.08% to 0.01% through HIP, with equiaxed grain formation also observed [15,16]. However, when the solution or annealing treatment is conducted at temperatures below 1100 °C, a small number of needle-like δ phases remain in the sample, along with incomplete recrystallization. Upon reaching a temperature of 1020 °C during solution treatment or annealing, the δ particles dissolve into the γ-matrix, leaving only carbides and oxides [17]. Subsequent aging or double aging after solution treatment typically leads to the formation of dense γ′ and γ″ nanoparticles. Research indicates that a solution treatment at 1020 °C for 15 min followed by aging at 720 °C for 24 h can completely eliminate Laves and δ particles while precipitating finely dispersed γ′ and γ″ nanoparticles [18].

Regarding the mechanical performance of additively manufactured IN718, most studies in the literature have focused on the tensile properties or fatigue at ambient temperature, as well as the impact of the post-heat treatments on these properties. For instance, the standard solution and double aging treatment have been found to yield a strength of 1250 MPa and an elongation of 16% [19]. When HIP is followed by a solution and double aging treatment, the strength increases to 1385 MPa with a ductility of 23.4%, where the ductility is higher compared to the standard solution and double aging [20]. Recent research has indicated that introducing a cold rolling step before heat treatment leads to equiaxed grains, resulting in a strength of 1600 MPa and an elongation exceeding 15% [21]. However, it should be noted that cold deformation can be challenging to implement for additive manufactured parts, particularly those with thin walls or complex shapes. The effects of various post-heat treatments on the microstructure and room-temperature mechanical properties have been extensively studied.

The literature on the high-temperature properties of the L-PBF (laser powder bed fused) IN718 alloy is relatively sparse compared to ambient temperature studies. For example, one study investigated the high-temperature wear behavior of as-built L-PBF IN718 up to 600 °C [22]. Xu et al. examined the creep performance at 650 °C, finding that heat-treated and HIP (hot isostatic pressing) specimens had significantly lower lifetimes (20–90 h) compared to hot-rolled specimens (200 h under similar conditions) [23]. Another study by Stephen reported that heat-treated IN718 samples exhibited strengths below 900 MPa at 600 °C [24]. Alexandra et al. systematically studied the effect of the process parameters on the high-temperature properties of the IN718 alloy at 650 °C, noting that the as-built samples generally showed strengths below 1000 MPa and exhibited mechanical anisotropy [25]. Ho investigated the impact of CoAl_2_O_4_ inoculants on the microstructures and high-temperature tensile properties, finding that double aging (aged at 720 °C for 8 h followed by 620 °C for 8 h) resulted in strengths around 987 MPa and elongation of 19.3% at 650 °C; the addition of CoAl_2_O_4_ slightly increased the strength to 1036 MPa with a decreased elongation of 16.7% [26]. 

The present study aims to investigate the effect of post-heat treatment on the microstructures and tensile properties of the L-PBF IN718 alloy at both ambient temperature and elevated temperatures. The microstructures of the as-built and heat-treated samples were systematically examined using SEM, EBSD, and TEM. The tensile properties of the samples were evaluated at both room temperature and specifically at 650 °C. Additionally, nanoindentation was performed at 650 °C to assess the creep resistance.

## 2. Materials and Methods

### 2.1. Sample Preparation

**Figure 1:**
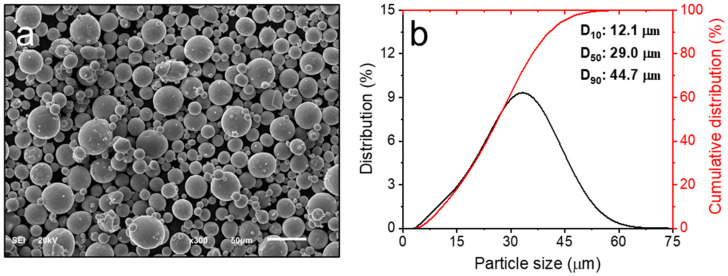
SEM image of the IN718 powder morphology (**a**) and size distribution (**b**).

Gas-atomized powders of the IN718 alloy were utilized in this study, sourced from Ninbo Zhonghe company (Ninbo, China). The chemical composition of the alloy powders was analyzed using inductively coupled plasma (ICP) spectroscopy, and the results are detailed in Table 1, meeting the ASTM B670 specifications [27]. Figure 1 illustrates the morphology and size distribution of the powders. The morphology was examined using a scanning electron microscope (TESCAN VEGA Compact, Brno, Czech Republic), while the size distribution was measured using a laser particle size analyzer (Horiba LA-960, HORIBA Scientific, Kyoto, Japan). The powders exhibited consistently spherical shapes, with average sizes (D_10_, D_50_, and D_90_) measured at 12.1 μm, 29.0 μm, and 44.7 μm, respectively.

**Figure 2:**
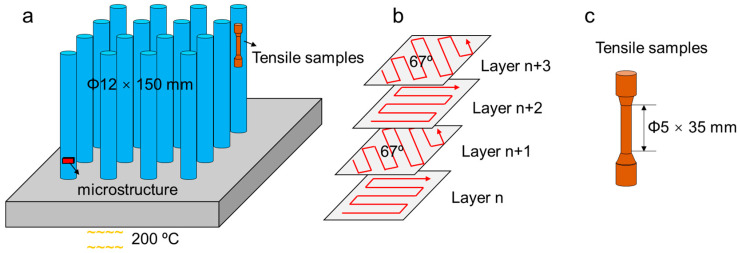
Schematics of sample preparation (**a**) scan strategy (**b**) and tensile samples (**c**).

The samples were manufactured using an EOS M290 equipped with a 400 W Yb fiber laser(EOS GmbH, Krailing, Germany). Bar samples having a dimension of ϕ12 × 150 mm were prepared, as depicted in Figure 2a. All the samples were fabricated under an argon-protective atmosphere to prevent oxidation during the processing. Based on prior optimization studies, the following process parameters were employed to ensure nearly dense samples: laser power of 260 W, scan speed of 1000 mm/s, layer thickness of 40 µm, and hatch spacing of 100 µm. The laser scanning strategy involved a stripe pattern with a rotation angle of 67° between successive layers, as illustrated in Figure 2b. The build substrate was preheated to 200 °C to mitigate residual stresses. The as-built samples were separated from the substrate by using wire-EDM. These samples are designated as “AB”.

### 2.2. Post-Heat Treatments

Two types of post-heat treatments were employed in this study: solution treatment, and solution treatment plus double aging. The as-built samples underwent initial solution treatment at 980 °C in a pre-heated furnace, held for 1 h, and then air cooled, designated as “ST”. Subsequently, the solution-treated samples were aged at 720 °C in a pre-heated furnace for 8 h, followed by a slow cooling to 620 °C over 8 h, and then air cooled. The cooling rate during the decrease from 720 °C to 620 °C was set at 50 °C/h. These aged samples are denoted as “STDA”. 

### 2.3. Microstructural Observation

For the microstructural analysis, the samples underwent mechanical grinding using silicon carbide sandpaper, followed by polishing with 5 µm and 1.5 µm diamond pastes. A final polish was conducted using a mixture of 20 vol.% H_2_O_2_ and 80 vol.% colloidal silica suspension (0.02 µm). The microstructural observations were carried out using Backscattering electron (BSE) imaging and Electron backscatter diffraction (EBSD). The BSE imaging was performed on a field emission scanning electron microscope (FESEM: TESCAN MIRA III, Brno, Czech Republic) operating at 15 kV. EBSD mappings were conducted using a FESEM (TESCAN Clara, Brno, Czech Republic) equipped with an EBSD detector (Oxford NordlysNano, High Wycombe, UK), operated at 20 kV with a step size of 1 µm and a working distance of 18 mm. The precipitate analysis of the aged samples was conducted using a transmission electron microscope (TEM, model: JEM 2100F, JEOL, Peabody, MA, USA) operating at 200 kV. The sample preparation for the TEM involved mechanical thinning to a 100 µm thickness, followed by final thinning using a precision ion polishing system (PIPS model 691, Gatan Inc., Warrendale, PA, USA).

### 2.4. Mechanical Testing

The samples for tensile testing had a gauge dimension of 5 mm in diameter and 35 mm in length, following GB/T 6397 [28] (Figure 2c). Tensile loading was applied parallel to the build direction. Room temperature tests were conducted at 25 °C using an MTS E45 tensile machine (MTS, Eden Prairie, MN, USA,) according to GB/T 228.1.2021 [29]. A constant speed of 1.5 mm/min was maintained using a contact extensometer, resulting in a strain rate of 1 × 10^−3^/s. For high-temperature tests at 650 °C, the samples were preheated in a furnace and equilibrated for 15 min to ensure the temperature uniformity, with a deviation of ±4 °C. Tests were conducted as per GB/T 228.2.2015 [30] using a constant speed of 0.5 mm/min and a corresponding strain rate of 0.33 × 10^−3^/s. The extensometer was removed after a deformation strain of 2%. The yield strength (YS) and ultimate tensile strength (UTS) were directly obtained from engineering stress–strain curves, while the total elongation to fracture (tEl) was determined by measuring the gauge length increments after fracture. Each test condition was repeated three times (Table 2), confirming the high repeatability (Figure S1).

Nanoindentation tests were conducted using a NanoTest Vantage system from Micro Materials Ltd., Wrexham, UK, equipped with a cubic boron nitride Berkevich indenter. Prior to testing, the samples were mechanically polished to achieve a mirror surface finish. Both the indenter and samples were maintained at 650 °C, monitored by thermocouples. During the testing, a fixed load of 50 mN was applied, reaching the preset maximum load within 5 s and was held for 600 s before reducing the load to 5 mN. The displacement and load measurements of the indenter were recorded with resolutions of 0.002 nm and 10 nN, respectively. To ensure the accuracy, multiple regions of each sample should ideally be tested. However, due to the rapid wear of the indenter at high temperatures, resulting in significant costs for replacement, two distinct regions of each sample were tested under identical loading conditions (as detailed in Table 2). The results showed a minimal variation between the indentation measurements from these different regions.

## 3. Results and Discussion

### 3.1. Microstructural Observation

**Figure 3:**
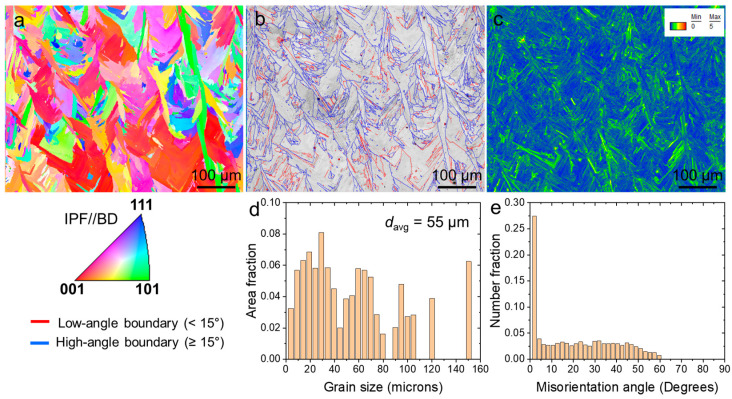
EBSD maps of the AB sample (**a**) IPF, (**b**) IQ + GB, (**c**) KAM, grain size distribution (**d**), and misorientation (**e**).

Figure 3 presents the EBSD maps illustrating the microstructural characteristics of sample AB. The predominant feature observed is a columnar structure typical of AM superalloys [14,18]. Melt pools with a wavy shape are clearly visible. The inverse pole figure (IPF) map in Figure 3a indicates that most of the grains are orientated with a [001]//build direction (BD). The sample exhibited a non-uniform distribution of grain sizes, with an average size of approximately 55 μm, as depicted in Figure 3d. In Figure 3b, the image quality (IQ) + grain boundary (GB) maps reveal that around 70% of the grain boundaries are high angles (≥15°), while low-angle grain boundaries are sparse. Figure 3e provides a quantitative representation of this distribution. The kernel average misorientation (KAM) map in Figure 3c illustrates the presence of numerous dislocations, particularly near the grain boundaries.

**Figure 4:**
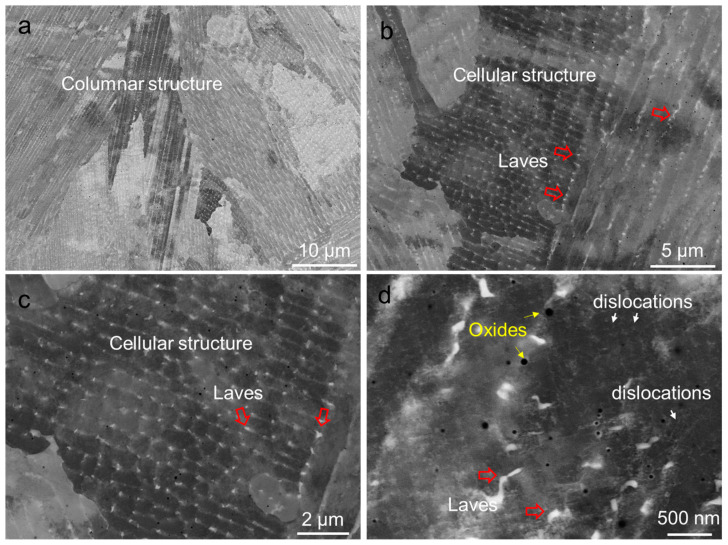
SEM BSE image showing the fine structures and substructures of the AB sample: (**a**) columnar structure, (**b**) cellular structure, and (**c**,**d**) substructures.

Figure 4 presents the BSE images illustrating the interior structure of the columnar grains in the sample. Both columnar dendrites and cellular dendrites are visible. Columnar dendrites have a thickness ranging from 1 to 2 μm, as depicted in Figure 4a, while cellular dendrites are smaller, approximately 0.5 to 1 μm in size, as shown in Figure 4b. Figure 4b,c highlight the presence of white nanoparticles at the boundaries of dendrites, indicated by arrows. These nanoparticles are identified as Laves phases and MC carbides, consistent with the findings from previous studies [31,32]. Figure 4d also shows the presence of oxides (black particles), marked by arrows, alongside individual dislocations (gray lines).

**Figure 5:**
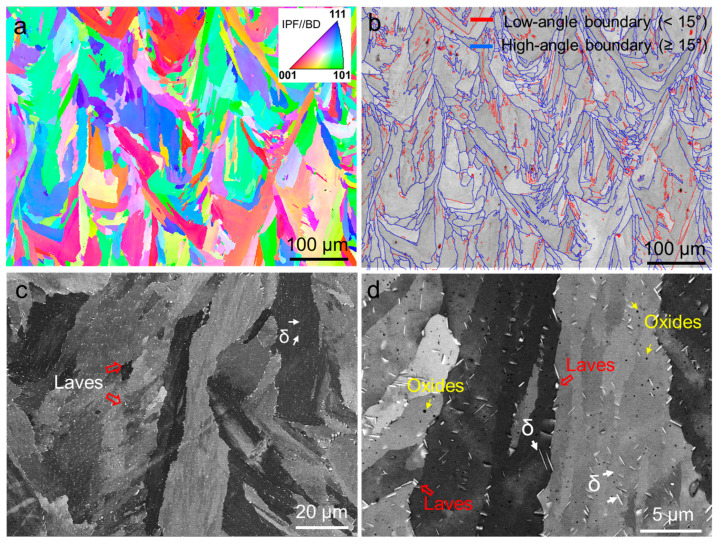
EBSD maps and SEM images of the ST sample: (**a**) IPF, (**b**) IQ + GB map, (**c**,**d**) BSE images.

Figure 5 illustrates the microstructures of sample ST. Despite undergoing solution treatment, a columnar structure remains evident, with a slight grain growth observed, as depicted in Figure 5a. A significant change in the grain orientation is notable compared to Figure 3a, indicating a shift in texture. Figure 5b shows the presence of a considerable number of low-angle boundaries. The interior microstructure exhibits substantial transformations: the disappearance of cell and plate structures, the dissolution of most of the nanoparticles into the matrix, and the homogeneous formation of fine needle-like δ phases within the matrix and along high-angle grain boundaries. Figure 5c highlights the presence of numerous intra-granular δ plates, approximately 1 to 2 µm in length. Additionally, Figure 5d reveals the presence of Laves phases and oxides, suggesting that the solution temperature was insufficient to fully dissolve these phases into the matrix.

**Figure 6:**
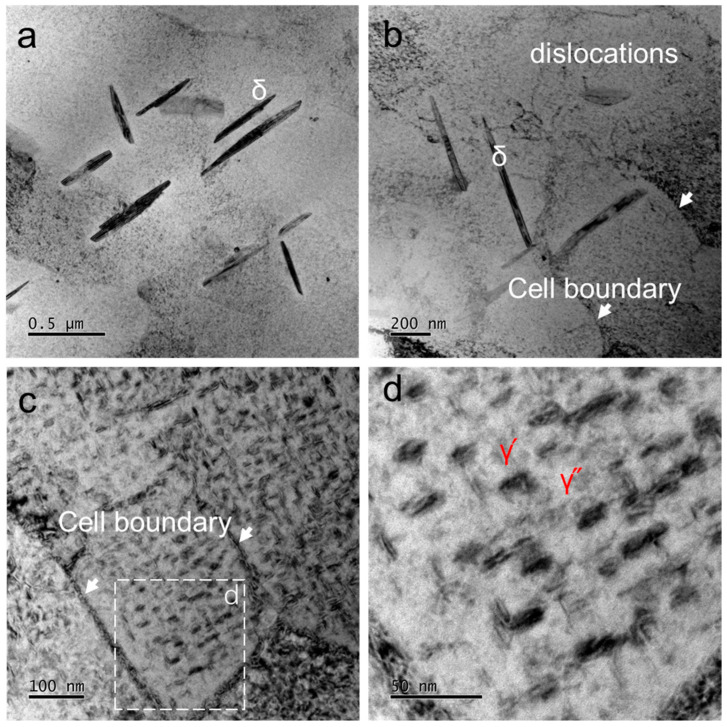
TEM images showing the fine precipitates and substructures of the STDA sample. (**a**) δ-phase, (**b**) substructures, (**c**,**d**) nanoprecipitates along the cell boundary and in the matrix.

Figure 6 displays the microstructures of the STDA sample observed using the TEM. Figure 6a clearly shows the presence of δ particles, which exhibited a length of approximately 0.5 μm and a thickness of 50 nm. Additionally, a small number of dislocations can be seen in the sample, as demonstrated in Figure 6b. The higher magnification images in Figure 6c,d revealed a fine dispersion of precipitates, identified as γ′ and γ″ phases formed during the double aging process. These γ′ and γ″ precipitates had a size of approximately 25 nm, resulting in a fine-grained microstructure with a uniform distribution of strengthening precipitates generated during the double aging treatment.

### 3.2. Mechanical Performance of the Samples at Room Temperature

**Figure 7:**
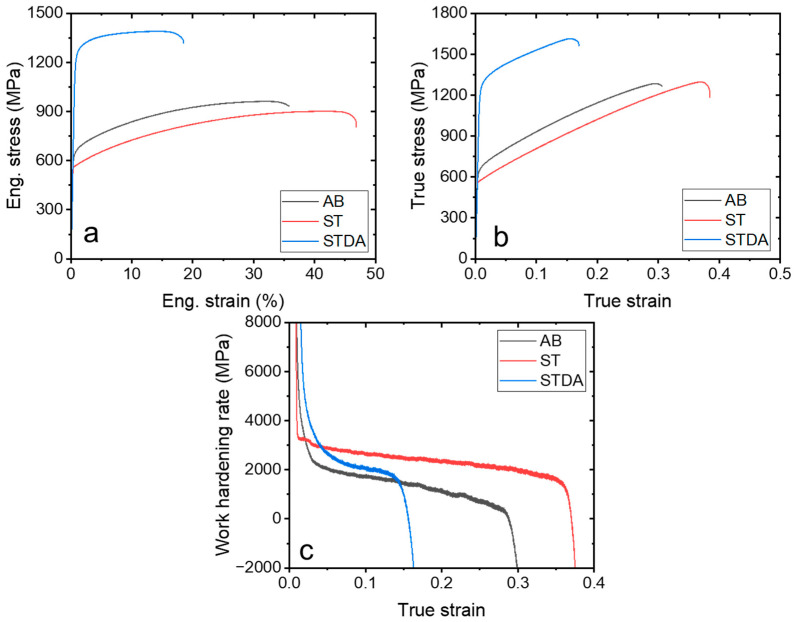
Tensile properties of as-built and heat-treated samples at room temperature: (**a**) engineering stress–strain curve, (**b**) true stress–strain curves, and (**c**) work-hardening rate.

Figure 7 presents the tensile stress–strain curves of samples AB, ST, and STDA tested at room temperature, with their corresponding properties summarized in Table 3. The stress–strain curve for sample AB exhibits significant plastic strain accompanied by pronounced work hardening. This sample demonstrates an ultra-high work-hardening ability (UTS-YS = 0.33 GPa, YS/UTS = 0.66) and good uniform elongation (>30%). The substantial strain hardening is primarily attributed to coarse γ grains (Figure 2) and where dislocation slips activate and propagate readily within the γ-matrix due to the sparse presence of particles or precipitates (Laves phase and oxide in Figure 4). This characteristic explains why the as-built IN718 alloy samples using L-PBF often display a low yield strength (typically < 700 MPa) and a very high ductility (tEl > 30%). Similarly, the ST sample demonstrates an excellent work-hardening ability, resulting in a uniform elongation of approximately 45%. The ST process reduces the dislocation density and particles further, enhancing the work hardening. Conversely, the subsequent double aging introduces finely dispersed nanoprecipitates (γ′ and γ″), which weakened the work-hardening ability (UTS-YS = 0.16 GPa, YS/UTS = 0.88). Consequently, the uniform elongation decreases to approximately 15%. This behavior is typical in precipitate-hardened Ni-based alloys, where the interaction between γ′ and γ″ nanoprecipitate and dislocations causes rapid initial hardening during the deformation. However, the dislocation accumulation saturates quickly during the subsequent straining, limiting the further work hardening capacity [33,34].

The AB sample shows a UTS of 967 MPa, a YS of 638 MPa, and a tEl of 36.6%, consistent with the findings from previous studies, as listed in Table 3. However, these room-temperature properties fall short of meeting the AMS 5383 standard [35] for cast IN718, particularly in YS, which should minimally be 700 MPa according to AMS 5383 [35]. Following the solution treatment, the samples exhibited a slight strength decrease (YS of 570 MPa) and improved ductility of 47.2%, attributed to the coarsening effect of the fine substructures induced by the solution treatment. Subsequent aging treatments are therefore necessary to strengthen the γ-matrix. In contrast, the STDA sample demonstrates a significantly higher strength (UTS of 1394 MPa and YS of 1229 MPa) and a tEl of 18.6%. These tensile properties notably exceed the reported data, as summarized in Table 3, and fully comply with the requirements specified by AMS 5662 [36] for wrought IN718.

### 3.3. Mechanical Performance of the Samples at an Elevated Temperature

**Figure 8:**
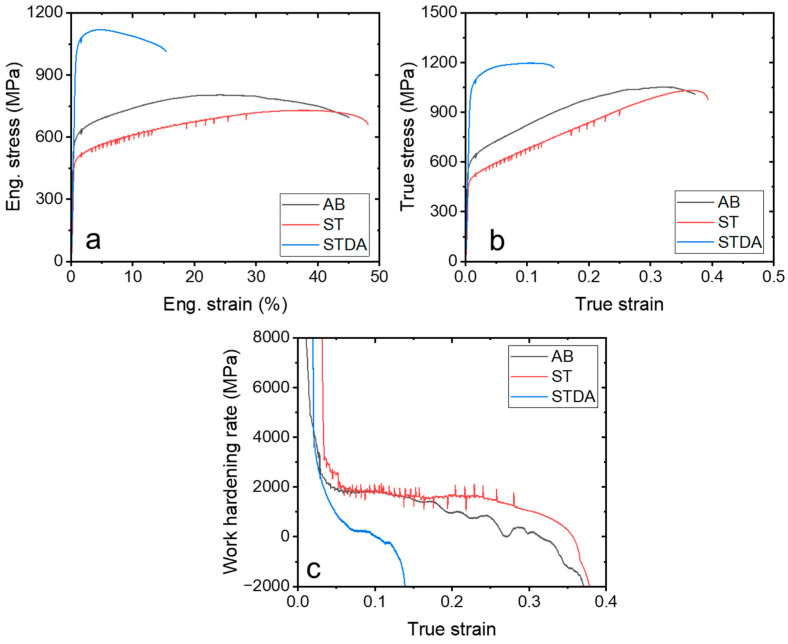
Tensile properties of as-built and heat-treated samples at 650 °C: (**a**) engineering stress–strain curve, (**b**) true stress–strain curves, and (**c**) work-hardening rate.

Figure 8 illustrates the tensile stress–strain curves of the AB, ST, and STDA samples tested at 650 °C, with their corresponding properties summarized in Table 4. Similar to the room temperature tests, significant strain hardening occurred in the AB and ST samples, resulting in a high uniform elongation: 22% for AB and 40% for ST. This work-hardening mechanism involves dislocation slips facilitated in the γ-matrix. In contrast, the STDA sample exhibited rapid necking followed by flow softening, leading to a reduced uniform elongation of only 4.6%. This flow softening phenomenon of IN718 has been noted in previous studies [2].

The AB sample displayed a UTS of 813 ± 8 MPa, a YS of 567 ± 13 MPa, and a tEl of 42.8 ± 8.3%, as detailed in Table 3. Although the yield strength is slightly lower than the reported values, the ductility significantly exceeds the reported data [25,26,37]. This discrepancy can likely be attributed to the substrate preheating at 200 °C during the fabrication, which reduces the dislocation density and residual stress [38,39]. Conversely, the ST sample exhibited a lower strength (UTS of 735 ± 13 MPa, a YS of 507 ± 21 MPa) and higher ductility (45.1 ± 6.4%) due to the absence of the substructures like cell structures and sub-boundaries (Figure 5). However, the high-temperature properties of the samples do not meet the requirements of AMS 5662 (wrought IN718) due to the insufficient strength, necessitating aging for alloy hardening in high-temperature applications. The STDA sample demonstrated a UTS of 1124 ± 6 MPa, a YS of 1025 ± 20 MPa, and an El of 12.3 ± 3.6%. These properties are comparable to the reported values [40,41] and conventional wrought IN718. 

**Table 4:** Tensile properties at 650 °C of the samples in this work and the literature.

Samples	UTS/MPa	YS/MPa	tEl/%	Ref.
AB	813 ± 8	567 ± 13	42.8 ± 8.3	This work
ST	735 ± 13	507 ± 21	45.1 ± 6.4	
STDA	1124 ± 6	1025 ± 20	12.3 ± 3.6	
AB	860	600	25	[25]
AB	987	800	19.3	[26]
AB	879	815	8.5	[37]
STDA	992	860	14.2	[40]
STDA	1100	-	12	[41]
Wrought 718	1000	862	12	[36]

### 3.4. Nanoindentation at an Elevated Temperature

**Figure 9:**
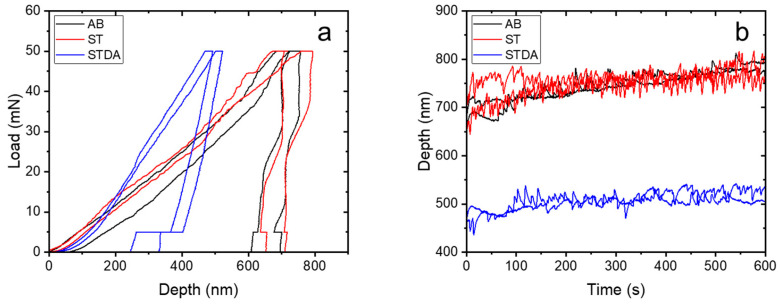
Nanoindentation results of the samples measured at 650 °C: (**a**) load–depth curve, (**b**) depth–time curves.

Figure 9 illustrates the load–depth and depth–time curves of the samples undergoing nanoindentation at 650 °C, with the corresponding data outlined in Table 5. Figure 9a specifically depicts the typical load–displacement curves for the three samples. Notably, the AB and ST samples exhibited a similar load–depth pattern, whereas the STDA sample demonstrated a distinct behavior, with a depth penetration that was only half of that observed in the AB and ST samples. As calculated from the load–displacement curves, Table 4 reveals the nanohardness and elastic modulus of the samples. Both the AB and ST samples displayed a nanohardness and elastic modulus of 3.0 GPa and 105 GPa, respectively. In contrast, the STDA sample exhibited significantly higher values, with a nanohardness of 6.3 ± 1.1 GPa and an elastic modulus of 139.5 ± 5.2 GPa. The increase in hardness at a high temperature is approximately twice that of the AB and ST samples, aligning with the observed high-temperature yield strengths in these samples. 

Figure 9b illustrates the depth–time curves of the samples, showing a rapid initial increase in depth penetration followed by a gradual rise. This behavior suggests an excellent creep resistance, as noted in recent research [42]. Particularly, the STDA sample maintains a nearly constant depth at around 500 nm, significantly lower than the AB and ST samples (~750 nm). This indicates a superior creep resistance under a constant high-temperature load compared to the other samples, attributed to the strengthening effect of the finely dispersed γ′ and γ″ nanoprecipitates within the γ-matrix, as previously discussed [23]. Moreover, previous studies have highlighted that L-PBF-manufactured IN718, after solution and double aging, exhibits an enhanced creep performance relative to its cast and wrought counterparts. This improvement is attributed to a higher volume fraction of γ′ and γ″ phases, which serve as primary strengthening agents [43].

In summary, the experimental findings of this study demonstrate that standard post-heat treatments have produced a specific microstructure comprising a substantial number of uniformly distributed δ-phase needles in the γ-matrix, along with a high-volume fraction of γ′ and γ′′ strengthening nanoprecipitates. This structure not only provides superior mechanical properties at ambient temperatures, comparable to those of wrought IN718 counterparts, but also enhances the high-temperature strength and durability. However, the optimal heat treatments are anticipated to further enhance the material’s strength and ductility. Additionally, as a superalloy commonly used for structural applications at elevated temperatures, future research should focus on evaluating the creep resistance and high-temperature fatigue evaluation of AM 718 under optimized heat treatments. 

## 4. Conclusions

In this study, microstructure analyses and tensile testing at ambient and elevated temperatures were performed on L-PBF-fabricated IN718, compared to different heat treatments. High-temperature nanoindentation was also conducted to indirectly assess the creep resistance. The following conclusions can be drawn:

1. The microstructure of the as-built L-PBF 718 alloy exhibits elongated columnar grains with cellular and columnar dendrites, both showing an ultrafine scale. A small amount of Laves phase and oxides are present in the as-built condition. The solution treatment did not significantly alter the columnar structures but eliminated the cellular and columnar dendrites. Instead, grain coarsening and partial recrystallization occurred, accompanied by the formation of a δ phase. Needle-like δ particles were uniformly distributed in the γ matrix or formed along the boundaries. The double aging subsequent to the solution treatment resulted in the formation of finely dispersed γ′ and γ″ nanoprecipitates.

2. The as-built samples demonstrated a moderate strength at both room temperature and 650 °C, primarily due to the presence of few precipitates. After the solution treatment, the samples exhibited a similar strength and ductility to the as-built ones because the strengthening effect of the fine δ particles may have been offset by the grain growth and reduced substructures. Both the as-built and solution-treated samples displayed tensile properties below the AMS requirements. However, after solution treatment followed by double aging, the samples showed significantly improved tensile properties. At room temperature, they achieved an ultimate tensile strength of 1394 MPa with an elongation of 18.6%. At 650 °C, they reached an ultimate tensile strength of 1124 MPa with an elongation of 12.3%. These properties, attributed to the finely dispersed strengthening γ′ and γ″ nanoprecipitates, are comparable to those of their wrought counterparts. 

3. Nanoindentation at high temperatures revealed that the as-built sample and solution-treated sample exhibited similar creep behavior, whereas the double aging samples showed a significantly improved creep resistance compared to the other two. Nanoindentation at high temperatures proves to be an effective method for indirectly characterizing the creep behavior of superalloys.

## Figures and Tables

**Table 1:** Chemical composition of the powders used in this study.

Element	Cr	Fe	Nb	Mo	Ti	Al	C	Mn	Si	Ni
Wt.%	19.2	19.7	5.4	3.2	1.1	0.5	0.05	0.2	0.01	Bal.

**Table 2:** Number of samples tested for tensile tests and nanoindentation used in this study.

Samples	Tensile at 25 °C	Tensile at 650 °C	Nanoindentation
AB	3	3	2
ST	3	3	2
STDA	3	3	2

**Table 3:** Room-temperature tensile properties of the samples in this work and in the literature.

Samples	UTS/MPa	YS/MPa	El/%	Ref.
AB	967 ± 6	638 ± 14	36.6 ± 1.2	This work
ST	904 ± 3	570 ± 10	47.2 ± 0.6	
STDA	1394 ± 4	1229 ± 5	18.6 ± 0.3	
AB	970	680	31.6	[19]
AB	995	698	33.2	[20]
AB	900	630	18	[21]
AB	950	650	32	[23]
AB	998	700	30.8	[26]
AB	915	844	26.3	[31]
STDA	1560	1240	11.6	[18]
STDA	1195	963	15.8	[19]
STDA	1187	1056	26.1	[24]
STDA	1144	978	28.6	[24]
STDA	1384	1173	10.7	[31]
Cast 718	862	758	5	[35]
Wrought 718	1276	1034	12	[36]

**Table 5:** Nanoindentation results of the samples measured at 650 °C.

Samples	Nanohardness/GPa	Elastic Modulus/GPa
AB	3.0 ± 0.5	105.5 ± 4.6
ST	3.0 ± 0.4	107.8 ± 3.7
STDA	6.3 ± 1.1	139.5 ± 5.2

## Data Availability

The raw data supporting the conclusions of this article will be made available by the authors on request.

## Supplementary Materials

The following supporting information can be downloaded at https://www.mdpi.com/article/10.3390/ma17153735/s1, Figure S1: Engineering stress–strain curves of the samples tested at 25 and 650 °C.
