# Supplementary figures and images for: Microstructures and High-Temperature Mechanical Properties of Inconel 718 Superalloy Fabricated via Laser Powder Bed Fusion
Source: Materials (Basel). 2024 Jul 28;17(15):3735. doi: 10.3390/ma17153735 (PMC11312763; doi:10.3390/ma17153735)

Supplementary figure

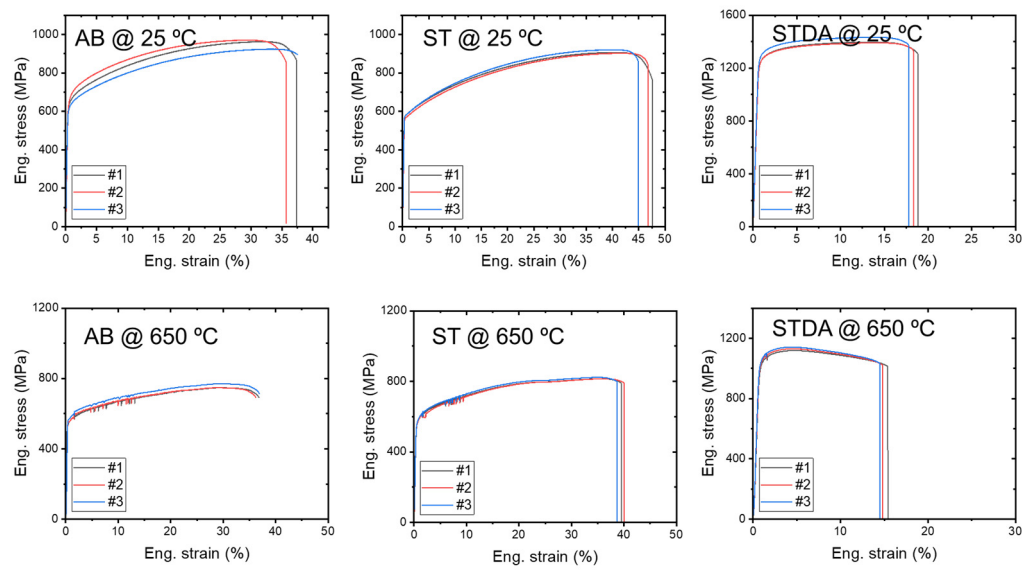

Figure S1. Engineering stress-strain curves of the samples tested at 25 and 650 °C.

Supplement: Supplementary file 1 [file materials-17-03735-s001.zip › materials-3107244-supplementary.pdf]
